# Complete Genome Sequence of Germline Chromosomally Integrated Human Herpesvirus 6A and Analyses Integration Sites Define a New Human Endogenous Virus with Potential to Reactivate as an Emerging Infection

**DOI:** 10.3390/v8010019

**Published:** 2016-01-15

**Authors:** Joshua Tweedy, Maria Alexandra Spyrou, Max Pearson, Dirk Lassner, Uwe Kuhl, Ursula A. Gompels

**Affiliations:** 1 Department of Pathogen Molecular Biology, London School of Hygiene & Tropical Medicine, University of London, London WC1E 7HT, UK; joshua.tweedy@lshtm.ac.uk (J.T.); maria-alexandra.spyrou@lshtm.ac.uk (M.A.S.); max.pearson@lshtm.ac.uk (M.P.); 2 Institute of Cardiac diagnostics (IKDT), Charite University, D-12203 Berlin, Germany; info@ikdt.de (D.L.); (U.K.)

**Keywords:** chromosomal integration, human herpesvirus, HHV-6, CiHHV-6, HHV-6A, CiHHV-6A, subtelomere, virus genome, telomeric repeats

## Abstract

Human herpesvirus-6A and B (HHV-6A, HHV-6B) have recently defined endogenous genomes, resulting from integration into the germline: chromosomally-integrated “CiHHV-6A/B”. These affect approximately 1.0% of human populations, giving potential for virus gene expression in every cell. We previously showed that CiHHV-6A was more divergent than CiHHV-6B by examining four genes in 44 European CiHHV-6A/B cardiac/haematology patients. There was evidence for gene expression/reactivation, implying functional non-defective genomes. To further define the relationship between HHV-6A and CiHHV-6A we used next-generation sequencing to characterize genomes from three CiHHV-6A cardiac patients. Comparisons to known exogenous HHV-6A showed CiHHV-6A genomes formed a separate clade; including all 85 non-interrupted genes and necessary cis-acting signals for reactivation as infectious virus. Greater single nucleotide polymorphism (SNP) density was defined in 16 genes and the direct repeats (DR) terminal regions. Using these SNPs, deep sequencing analyses demonstrated superinfection with exogenous HHV-6A in two of the CiHHV-6A patients with recurrent cardiac disease. Characterisation of the integration sites in twelve patients identified the human chromosome 17p subtelomere as a prevalent site, which had specific repeat structures and phylogenetically related CiHHV-6A coding sequences indicating common ancestral origins. Overall CiHHV-6A genomes were similar, but distinct from known exogenous HHV-6A virus, and have the capacity to reactivate as emerging virus infections.

## 1. Introduction

Uniquely for human herpesviruses, both human herpesvirus 6A and B, HHV-6A and HHV-6B, have forms integrated into the germline of human chromosomes in approximately 1% of human populations examined [1,2,3,4]. This integration involves homologous recombination between common telomeric repeat sequences present towards the ends of both virus genomes and the telomeres of human chromosomes, and leads to integration at the sub-telomere adjacent to the final repeats at the host chromosome termini. The resulting addition of over 150 kb from the virus genome gives an “inherited” endogenous form, CiHHV-6.

Since integrated genomes are present in every nucleated cell, quantification by real time DNA PCR can show this as abnormally high “viral loads” confounding infectious virus diagnostics [3]. In blood DNA, CiHHV-6 DNA “loads” appear significantly higher than even viremic children with primary infections [5]. Moreover, if the genomes are intact, there could be gene expression from over 85 viral genes in each cell as well as the potential for reactivated infectious virus [6,7]. Ramifications for health are beginning to be evaluated and crucial to this is an understanding of the relationship between exogenous HHV-6 virus and germline-integrated CiHHV-6 genomes.

In addition to the now well-established data regarding germline integration, there may also be somatic integration. *In vitro* evidence showed telomeric integration of HHV-6A/B genomic DNA in the absence of detectable viral episomes and led to the proposal that viral telomeric integration in somatic cells may be a strategy for establishing quiescent latency in contrast to the episomal latency characteristic of other human herpesviruses [6]. While such somatic cell genome integration has yet to be demonstrated *in vivo* for HHV-6A/B, germline cell integration has clearly been shown *in vivo*, that it can be inherited and results in a vertical congenital “infection” [1]. Somatic and germline integration could be intrinsically linked, but there are marked differences. Unlike somatic cells, germline cells actively maintain the telomere. In exogenous virus genomes the DR-L and DR-R, 8 kb each, are the left and right direct repeats at the end of the 143 kb UL, long unique region, of the virus genome, as shown for reference HHV-6A, strain U1102 [8], with the DRs bounded by “imperfect” and “perfect” repetitions of the hexameric human telomeric repeats which may mediate integration [9]. Initial analyses of several integration sites in CiHHV-6A and B, indicated the structure, telomere-(DR-L)-UL-(DR-R)-subtelomere, [6,10,11] (Figure S1) with terminal packaging signals deleted. Further analyses of CiHHV-6B integration in the subtelomere region showed additional adjacent DR regions in four patients [11]. Unlike the telomeric termini with perfect telomeric hexameric repeats, the subtelomere region has complex degenerate repeat structures, genes and gene expression, together with active recombination [12,13,14]. Therefore virus genome integration in this region in the germline may permit virus gene expression, reactivation by genome replication or possibly virus formation. This may affect foetal or infant development as well as generation of the immune system and its regulation.

The clinical outcomes of germline integration are beginning to be assessed. In infants with CiHHV-6A/B, studies showed negative effects on mental development, after earlier diagnoses as congenital “infection”, which are primarily via inherited endogenous virus genomes, although there is preliminary evidence for transplacental infection from CiHHV-6A/B mothers [1,15,16]. In a child with X-linked severe combined immunodeficiency disease and CiHHV-6A, evidence for reactivation was coincident with symptoms of thrombotic microangiopathy and gastrointestinal bleeding [17]. In adults, recent studies show a specific subset of cardiovascular disease patients with recurrent disease linked to CiHHV-6A/B [18], with a further case report also showing heart failure in a neonate with CiHHV-6A/B [19], and recent cohort screens demonstrating links with angina [20]. This shows CiHHV-6A/B effects may originate from infancy, and could result in chronic states in the adult, therefore it is important to understand the origins, distinctions and interactions with infectious virus of these integrated, inherited genomes.

Our previous studies on a Czech/German CiHHV-6A/B cohort showed greater prevalence and diversity for CiHHV-6A. This was shown by comparing sequences from four loci from 44 CiHHV-6A and CiHHV-6B patients in relation to known HHV-6A and B reference strains. One of the loci could be compared to sequence available in over 80 clinical HHV-6A and B strains globally also showing greater diversity in CiHHV-6A [21]. Moreover, we showed gene expression in four patients and using deep sequencing analyses of three genes from integrated genomes demonstrated that a CiHHV-6A cardiovascular disease patient had superinfection with circulating HHV-6A virus [21]. Where there was exogenous virus superinfection, we detected virus gene expression derived from the integrated genome, suggesting virus superinfection reactivated expression from the integrated-genome; there was no gene expression in another CiHHV-6A patient without superinfection [21]. In contrast, in four CiHHV-6A/B patients with neurological disease, analyses of one gene showed gene expression from superinfecting HHV-6A/B, demonstrating susceptibility to infection with exogenous virus [22]. CiHHV-6A reactivation or tolerance to exogenous virus superinfection may occur in different patients, but since the studies were only on selected loci, there could be differences at other regions of the genomes and the intactness or capacity of the CiHHV-6 genome to reactivate was not known. Therefore, CiHHV-6A appears different from circulating HHV-6A and in order to distinguish their effects, a better understanding of their genetic relationships is required and improved tools for their diagnoses are needed.

In this report we sought to further investigate the relationship between CiHHV-6A and exogenous HHV-6A by characterising the complete genome of CiHHV-6A, its coding complement and telomeric integration sites. The results show distinctions of the integrated-genomes from circulating virus, indicate ancestral origins at chromosome 17p integration, and demonstrate potential for emergence as infectious virus or sources for divergent viral gene expression in the affected host.

## 2. Materials and Methods

### 2.1. Patient and Virus DNA

DNA was extracted from European patients with germline chromosomal integrated CiHHV-6A or CiHHV-6B genomes as described previously [23]; the DNA was extracted directly from peripheral blood leukocytes from European patients with haematological disorders who were screened for CiHHV-6A/B, and were malignancy or inflammatory disease patients in the Czech Republic or from cardiac patients in Germany [23].

### 2.2. Sequence Accession Numbers from Reference Exogenous Virus

For comparisons to exogenous HHV-6A/B all available complete genomes were analyzed and their accession numbers from previous studies listed here. HHV-6A, strain U1102 and AJ, derived from Uganda and Gambian human immunodeficiency virus infection and acquired immune deficiency syndrome (HIV/AIDS) patients and further isolated and characterized in the UK [8,24,25], emb X83413.1, complete genome, and gb KP257584, respectively; strains GS from patients with hematologic disease in USA, (GS1) KC465951.1 [26], and (GS2) KJ123690.1. HHV-6B, strain Z29 and HST, complete genomes gb AF157706.1 and dbj AB021506.1, from Democratic Republic Congo HIV/AIDS patients and Japanese ES, exanthem subitum, patients, respectively [27,28]. Accession numbers from prototype human herpesviruses are shown in Figures in the Results. Accession numbers from new sequences from CiHHV-6A genomes from this study are in the next section.

### 2.3. Illumina Sequencing the CiHHV-6A Genomes and Sequence Assembly

Long range PCR was used to generate overlapping amplicons, 1–7 kb in length, across the entire CiHHV-6A genomes using primers based on reference HHV-6A strains U1102, GS, and AJ genome sequences as described [23]. Thirty-six overlapping PCR amplicons were generated using GoTaq Long PCR mastermix (Promega, Southampton, UK) and nuclease-free H_2_O (Sigma-Aldrich, Gillingham, UK) with thermocycling using a hot start 95 °C for 2 min, then 35 cycles of 95 °C for 20 s, 59 °C for 30 s, 70 °C for 6 min, and a final elongation step of 72 °C for 10 min. Amplicons were separated on 0.7% agarose gels, then purified using the Wizard SV gel and PCR clean-up kit (Promega). Next, equimolar pooled amplicons were sheared to an average size of 200 bp using an E210 focused-ultrasonicator (Covaris, Brighton, UK). The sheared fragments were then purified using Agencourt AMPure XP beads (Beckman Coulter, High Wycombe, UK) followed by end repair, dA-tailing, adapter ligation, and PCR enrichment using the NEBNext DNA library prep master mix set for Illumina together with multiplex oligonucleotides (New England Biolabs, Hitchin, UK). The post-reaction clean-up steps used Agencourt AMPure XP beads. The prepared libraries DNA quality and quantification were then assessed using the Agilent high sensitivity DNA kit (Agilent, Wokingham, UK) and a Qubit 2.0 fluorometer (Invitrogen, Paisley, UK). Denatured, indexed DNA libraries were then subjected to 2 × 150 bp paired-end sequencing utilising the MiSeq v2 reagent kit then run on an Illumina MiSeq (Illumina, Little Chesterford, UK).

The resultant raw-sequence data quality was first assessed with FastQC (Babraham Bioinformatics, Cambridge, UK). The Fastq file reads had adapters removed and quality trimming using a phred score of 33 and minimum length of 100 bp with trimmomatic version 0.32 [29]. All oligonucleotide primer sequences were removed. The trimmed reads were then mapped to both the HHV-6A strain U1102 reference, GS and AJ genomes [8] (Accession X83413.1, Ref Seq NC_001664.2) [23,26] with the BWA-MEM alignment algorithm and SAM tools [30,31]. The average read coverages were calculated using GATK DepthOfCoverage [32] and alignment qualities assessed using Qualimap [33]. For variant calling we used both a SAMtools mpileup, BCFtools, vcfutils varFilter pipeline [34] and GATK UnifiedGenotyper [32]. For mapping quality scores >25, Bcftools was utilized to variant call. Single nucleotide polymorphisms (SNPs) were then filtered using vcfutils varFilter with minimum and maximum read-depths adjusted to 10 and twice the average read depth, respectively. For *de novo* assembly, a VelvetOptimiser, Velvet [35], ABACAS [36] pipeline was used for assembly optimisation. For reference mapping contigs were ordered using references genomes from HHV-6A strains U1102, GS (KC465951.1) and AJ, together with manual adjustments using Artemis [37,38].

The Rapid Annotation Transfer Tool (RATT) was used to generate annotations [39] using the reference HHV-6A strain U1102 while incorporating subsequent updated annotations and GeneMark predictions [8,26,40,41]. Using both *de novo* and reference genome mapping mean read depths coverage of UL for CiHHV-6A 2284, 5055 and 5814 genomes were 1907, 19,307, and 4023, respectively. Aside from small repetitive regions in R2 and R3, which could not be resolved by Illumina sequencing, the UL was 100% covered for CiHHV-6A 2284 and 98% for 5055 and 5814. The repetitive regions in the DRs were more extensive and sequence divergence greater, therefore solely compiled *de novo* giving 40%, 83%, and 65% coverage with respect to reference (U1102) for CiHHV-6A 2284, 5055 and 5814, respectively. A template DR was constructed combining all fastq reads, then remaining gaps filled by Sanger sequencing to derive the DR with 100% coverage for 2284. The complete 2284 genome was then reconstructed with the cognate DR and UL sequences.

The new sequences from CiHHV-6A in this study are assigned Genbank accession numbers as filed KT895199-211.

### 2.4. Chromosomal Integration Site PCR Amplification

The CiHHV-6A chromosomal integration sites were amplified using PCR with primers (synthesized by Sigma-Aldrich) specific for the adjacent sequences at the subtelomere of chromosome 17p (5′ AACATCGAATCCACGGATTGCTTTGTGTAC 3′) and HHV-6 DR-R (5′ CATAGATCGGGACTGCTTGAAAGCGC 3′) [6,42]. GoTaq green mastermix (Promega,), nuclease-free H_2_O (Sigma-Aldrich) was used with thermocycling conditions of 94 °C for 5 min, then 40 cycles of 94 °C 15 s, 59 °C for 30 s, 72 °C for 5 min, with a final elongation step at 72 °C for 10 min. The 1.5 kb PCR products were separated and purified from 1% agarose gels using the Wizard SV gel and PCR clean-up kit (Promega,) followed by Sanger sequencing using the amplification primers (Source Bioscience, Nottingham, UK).

### 2.5. Multiple Alignments and Phylogenetic Analyses

Multiple alignments of nucleotide and encoded amino acid sequences were performed in MEGA5 using MUSCLE [43]. Phylogenetic trees for nucleotide and encoded amino acid sequences were built using the Maximum Likelihood method with all positions containing gaps and missing data eliminated and the tree constructed with the Bestfit model giving highest log likelihood produced using MEGA5 [43]. The nucleotide sequence trees were constructed using the General Time Reversible Model with gamma distribution and close neighbor interchange, allowing for invariant sites, and checked with 1000 bootstrap replicates.

For distance measurements, individual or catenated nucleotide sequences of conserved or divergent genes were translated then aligned using MUSCLE, then back-translated and trimmed for alignments, followed by phylogenetic trees constructed using maximum likelihood analyses in MEGA using the Bestfit model then pairwise distance measured.

## 3. Results

### 3.1. Genomic Analyses of CiHHV-6A

In order to understand the ancestral origins of CiHHV-6A a meta-analysis on geographic prevalence was conducted on all available studies of CiHHV-6 which had genotyping. Results were separated into CiHHV-6A and CiHHV-6B, showing 0.2% and 0.4% respectively, in screens of over 19,000 individuals identifying 115 with CiHHV-6A or B. There were indications of regional differences, with CiHHV-6A higher in Europe, 0.3%, compared to Japan, 0.04% (Table S1). Furthermore, our previous studies showed European CiHHV-6A sequences were more divergent than CiHHV-6B suggesting earlier origins [21]. Therefore, European CiHHV-6A genomes were investigated further here.

Three CiHHV-6A genomes were selected for further analyses and were from patients in the European (Germany) cohort who had persistent cardiovascular disease [18,44]. These could be from HHV-6A/B superinfection or CiHHV-6A/B reactivation. These samples had sufficient DNA available from blood collected during a recurrent disease episode. These were characterized further using our amplicon based target enrichment methods [21,23]. We previously compared this method to solution hybrid selection (Agilent SureSelect, Santa Clara, CA, USA) target enrichment methods in resequencing HHV-6A reference strain U1102 and determining the complete genome sequence of HHV-6A strain AJ [23,45]. The amplicon-based methods were used here as they allowed determination of sequences where there were potentially unknown reference sequences and could resolve the more divergent DR region. Resultant integrated CiHHV-6A genomic sequences were analyzed in comparisons to known exogenous HHV-6A genomic sequences including strains AJ, U1102, and GS.

#### 3.1.1. Phylogenetic Relationships between CiHHV-6A and HHV-6A

We determined the complete genome sequence of CiHHV-6A 2284. All identified putative open reading frames were identified homologous to the 85 annotated in the reference HHV-6A U1102 virus genome. There was no evidence for disrupted defective genes. These were compared to coding sequences derived for CiHHV-6A 5055 and 5814. Alignments and phylogenetic reconstructions were made using catenated core conserved genes. These conserved genes were used previously to examine phylogenetic relationships between human herpesvirus species [46].

**Figure 1:**
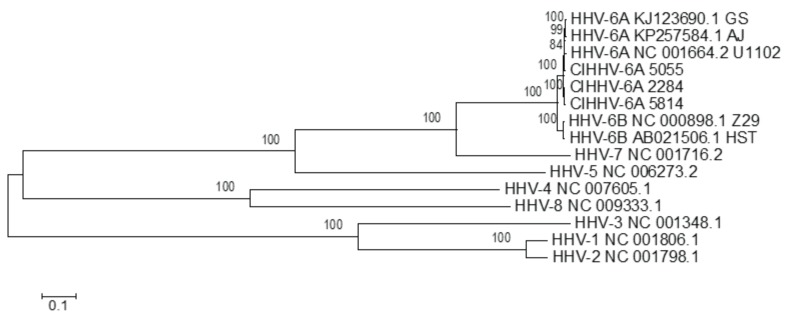
Phylogenetic analyses of CiHHV-6A compared to human herpesvirus representative of *herpesviridae* species in alpha, beta, and gamma herpesvirus sub-families. Maximum likelihood method was used in Mega 5.1 using core conserved genes to compare the relationship of CiHHV-6A 5055, 2284, and 5814 genomes to betaherpesviruses including the *Roseolovirus* genera of HHV-6A, HHV-6B, HHV-7, together with Human cytomegalovirus (HHV-5); gammaherpesviruses Epstein Barr virus (HHV-4) and Kaposi’s sarcoma associated herpesvirus (HHV-8); alphaherpesviruses Herpes Simplex Virus (HSV) type 1, HSV-1 (HHV-1), and HSV-2 (HHV-2). The reference sequences accession numbers used are shown in the figure. Core genes included homologues of capsid triplex subunit 1 (U29), small capsid protein (U32), large tegument protein (U3), large tegument binding protein (U30), cytoplasmic egress tegument protein (U71), cytoplasmic egress facilitator-1b (U44), glycoproteins gB, gL, gM (U39, U82, U72), multifunctional expression regulator (U42), DNA polymerase catalytic subunit (U38), DNA polymerase processivity subunit (U27), helicase-primase RNA polymerase subunit (U43), helicase primase subunit (U74), single-stranded DNA-binding protein (U41), alkaline deoxyribonuclease (U0), uracil DNA glycolase (U81), ribonucleotide reductase large subunit (U28), capsid transport nuclear protein (U36), DNA packaging terminase subunit 2 (U40), terminase binding protein (U35), nuclear egress membrane protein (U34), and nuclear egress lamina protein (U37). Bootstrapping (1000) shows percentage of trees where taxa cluster together and scale shows branch lengths measured in number of substitutions per site.

We used this approach here in order to investigate the relationship of the CiHHV-6A endogenous genomes to known exogenous herpesvirus genomes. The comparisons here show CiHHV-6A genomes tightly linked to known exogenous HHV-6A, but also distinct (Figure 1).

**Figure 2:**
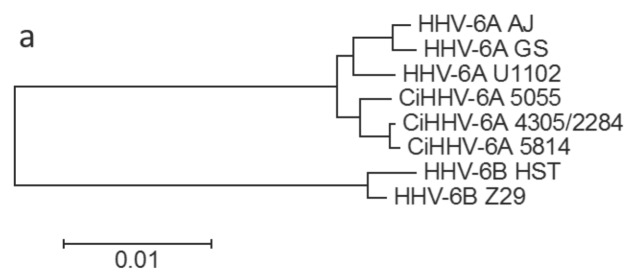

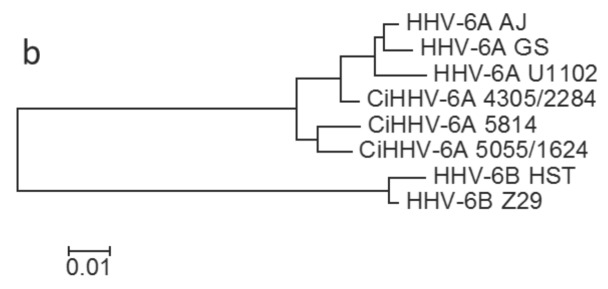
Phylogenetic tree analyses of CiHHV-6A and *Herpesviridae*, conserved core and variable genes. Maximum likelihood method was used in Mega 5.1 using Bestfit model of General Time Reversible with gamma distribution (methods) with maximized amino acid alignment followed by back-translation and phylogenetic reconstruction. (**a**) Conserved core genes were compared showing relationships. These included 14 genes encoding mainly HHV-6A/B conserved structural and DNA replication components and included U31-39, U42-44, U74, U81-82; (**b**) Variable genes as defined in Table 1, included 16 genes and were compared to homologues in genomes of strains of HHV-6A/B virus. Bootstrapping (1000) shows percentage of trees where taxa cluster together were >95% for all nodes. The scales shows branch lengths measured in number of substitutions per site. HHV-6B was used as an out-group to analyze relationships between HHV-6A and CiHHV-6A.

We next examined in more detail the phylogenetic relationship of CiHHV-6A to known exogenous HHV-6A to investigate whether there are possible distinct clades and their origins. In order to determine overall relationships and take account of possible different evolutionary rates in conserved or non-conserved genes [47,48], or in integrated/non-integrated genes, genes were clustered into groups of conserved and divergent genes. Phylogenetic analyses showed that the CiHHV-6A catenated genes clustered basal with respect to known exogenous HHV-6A in the divergent gene phylogeny. Using HHV-6B virus genomes as a relative outgroup, comparisons of conserved genes show clustering of the CiHHV-6A integrated-genome genes separate from that of the exogenous HHV-6A virus reference genomes (Figure 2a); while comparisons of the variable genes show further divergence and an ancestral root basal to the HHV-6A ancestral node. There was mixed branching between the endogenous and exogenous virus genomes in that the relative branching order was different between conserved and variable gene sets (Figure 2a,b, see 2284/4305) and indicative of recombination.

**Table 1:** Pairwise comparisons to HHV-6A U1102 show CiHHV-6A variable genes+.

Gene	HHV-6A		CiHHV-6A			HHV-6B	
	AJ	GS	2284	5055	5814	Z29	
	%	%	%	%	%	%	Gene Function/Homologue, References
*DR1* *	96.7	96.9	95.9	96.8	96.7*i*	88.9	Putative DNA directed RNA polymerase Z29
*DR6* *	95.5	96.1	96.6	96.3	95.9	87.8	DR6B binds p41 DNA processivity factor U27, inhibits replication, G2/M arrest [50,51]
*U11*	97.9	97.8	98.0	98.0	97.8	89.2	Tegument phosphoprotein, pp100 major antigen (HCMV UL32)
*U13*	99.7	99.7	99.7	97.5	97.5	96.2	
*U14*	99.6	99.7	99.6	96.5	96.0	90.5	Virion tegument protein (HCMV UL25/35), P53 interaction cell cycle [52]
*U15* *	97.1	97.4	97.2	97.2	99.3	94.0	
*U19*	97.9	97.7	97.9	97.7	98	94.2	IE-B protein (HCMV US22 gene family, UL38)
*U47*	98.0	98.4	97.5	97.5	97.5	92.9	Membrane glycoprotein gO complexes with gH/gL (HCMV gO U74)
*U54*	99.3	98.8	97.2	97.2	97.2	87.5	Virion transactivator, (pp65, HCMV UL82/83) U54A activates NFAT [53]
*U65*	99.5	99.6	97.4	97.5	97.5	92.8	Tegument protein (provisional HCMV UL94, HSV UL16)
*U71*	99.2	99.2	99.2	95.3	95.3	92.6	Myristylated tegument protein; position HCMV pp28K (HSV UL11)
*U79* *	98.5	96.6	96.5	96.8	96.3	89.8	DNA replication provisional, includes U80, (HCMV UL112/113,P34)
*U86* *	98.3	97.6	97.9	95.1	94.3	85.5	IE2, IE-A protein; includes U87; (HCMV IE2 UL122), R1 repeats
*U90* *	97.8	97.6	97.4	97.5	97.3	83.8	IE1, IE-A transactivator; includes U89; (position HCMV IE1)
*U95*	97.8	97.6	97.2	96.5	95.7	84.0	(HCMV US22 gene family, position MCMV IE2), GRIM-19 interaction, mitochondria [54]
*U100* *	98.0	97.5	97.0	97.1	97.0	90.3	Membrane glycoprotein gQ complexes with gH/gL binds CD46
Mean	98.2	98	97.6	97.0	96.8	90.5	

+Genes were translated, aligned by Muscle, phylogenetic analyses of nucleotide sequence using Bestfit model with maximum likelihood analyses (Tamura-3-parameter with Gamma distribution, HK or GTR) and then pairwise distance analyses with <98% cut-off compared to CiHHV-6A genes using Mega5.1 as compared to orthologues in HHV-6A/B. * HHV-6A defined splice donors and acceptors as annotated used to generate spliced products. *i* = partial, exon2 DR6. Shaded scores show <97% cut off. Functions/homologues/references from genome annotations and cited. Brackets show homologues in other herpesviruses.

#### 3.1.2. Divergent Genes in CiHHV-6A

Comparisons were made between available genomes from exogenous HHV-6A/B virus strains and the CiHHV-6A endogenous integrated virus genes. The SNP density was 10–20 SNPs per kb giving a mean diversity between reference and known HHV-6A virus genomic strains of 1%–2%. This cut-off was confirmed by analyses of 56 strains from clinical tissue characterized by direct sequencing of two main variable genes [49] as well as a further variable gene in over 80 strains [21]. Therefore a cut-off of 2% was applied to define divergent genes in the CiHHV-6A genome. The CiHHV-6A genomes had 16/85 genes divergent from HHV-6A strains with 2%–6% variation, with a SNP density of 20–60 SNPs per kb, even after considering spliced genes as summarized in Table 1. There were 4–8 genes with over 3% variation in CiHHV-6A genomes compared to only two restricted to the DR repeats in HHV-6A (Table 1).

These divergent gene functions included all the variable genes in HHV-6B that distinguish this virus from HHV-6A. These gene divergences between HHV-6A and HHV-6B had supported their classification as separate species, as reviewed [27,55,56] (Table 1). Group taxa distance measurements show the CiHHV-6A genes differentiate from known exogenous HHV-6A. Though closer to HHV-6A, CiHHV-6A genes are intermediate to HHV-6B (Table 2).

**Table 2:** Distance and diversity measurements in group taxa analyses.

Taxa Comparisons	HHV-6A	CiHHV-6A	HHV-6B
*Mean distance*			
HHV-6A	0.015		
CiHHV-6A	0.029	0.023	
HHV-6B	0.131	0.128	0.011
*Mean diversity*			
HHV-6A	0.017		
CiHHV-6A	0.025 (0.246)	0.019	
HHV-6B	0.071 (0.844)	0.085 (0.801)	0.013

Mean distance or diversity (and coefficient differentiation) were measured on catenated variable genes from Table 1 (methods); taxa groups HHV-6A: strains U1102, GS, AJ; HHV-6B: strains Z29, HST; CiHHV-6A: 2284/4305, 5055, 5814.

The 16 genes did not show uniform differences; both 5055 and 5814 were more divergent than 2284/4305. The 16 genes included abundant tegument proteins, which are CD4 and CD8 T-cell targets as well as membrane envelope glycoprotein targets for neutralizing antibody. The functions include those that control gene expression, cell cycle, and immune regulation. Additionally at this site the transcriptional regulator, immediate early IE1 gene, U90, also had the deletion which renders the virus susceptible to interferon [57] in all the CiHHV-6A strains. Further, U54 retains the motif that distinguishes inhibition of transactivation of the nuclear factor of activated T-cells (NFAT) in HHV-6B from HHV-6A [53]. These analyses show CiHHV-6A genomes are divergent at a set of genes which encode products involved in gene regulation, host infection, cellular tropism, and immune regulation, all previous markers for herpesvirus speciation. These could represent integration of an ancestral HHV-6A virus genome or effects of repeated integration/excision cycles.

#### 3.1.3. Identification of Exogenous HHV-6A “Superinfection” in CiHHV-6A Patients by Deep Sequencing

We previously showed by deep sequencing that 2284/4305 was superinfected by exogenous HHV-6A strains. This was by analyses of the variable chemokine gene *U83* and polymerase gene *U38* [21]. We extended this using the divergent gene set here, with an example given in Table S2 for *U54*. There were 51 SNPs distinguishing CiHHV-6A from exogenous HHV-6A strains. The CiHHV-6A sequences were identical between the three patients. In contrast, the superinfecting virus was identified by 37 SNPs shared with known exogenous virus. 36/51 SNPs were non-synonymous coding changes including 26/37 superinfecting virus SNPs. This showed CiHHV-6A 2284 had exogenous virus “superinfection” of between 15% to 30%, sharing SNPs from HHV-6A U1102, GS and AJ. CiHHV-6A 5814 had only low level of exogenous virus SNPs, 2%–4%, at three locations; while 5055 did not have any evidence for exogenous infection. Both 2284/4305 and 5814 were CiHHV-6A patients with recurrent cardiovascular disease [18,44].

### 3.2. Characterization of a Common Integration Site at Chromosome 17p

**Figure 3:**
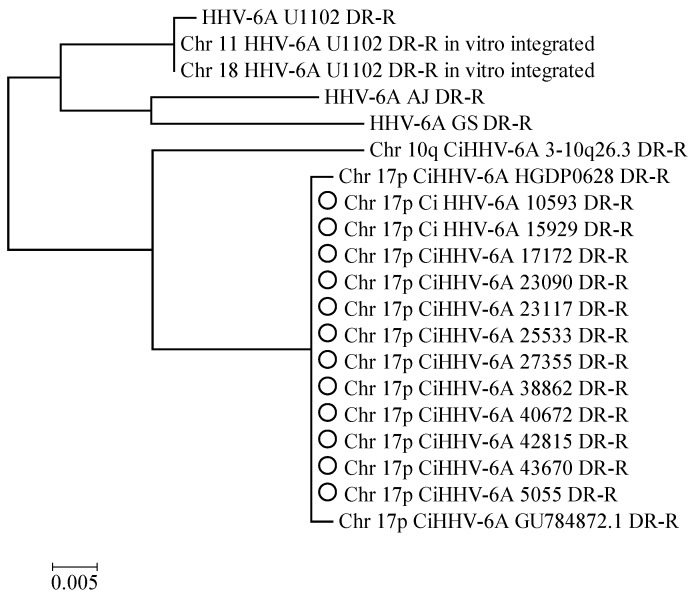
Phylogenetic analyses show the DRR adjacent sequence compared between CiHHV-6A integrated at 17p compared to other chromosomes, with 100% bootstrap (1000×) support main nodes.

Previously, we had determined the integration site at chromosome 17p for 12 CiHHV-6A patients by PCR amplification using specific primers [21]. Therefore, over a quarter of the CiHHV-6A patients cohort we had initially characterized [21], had integration at this site showing a favored or ancestral site here. In order to investigate relationships of endogenous genomes in 17p, we determined the complete sequence of the integration site and analyzed the relationships between the repeat structures and also the coding sequences from the endogenous genomes. This showed identical virus DR-R regions and subtelomeric 17p unique sequences adjacent to the integration site, indicating shared origins (Figure 3).

**Figure 4:**
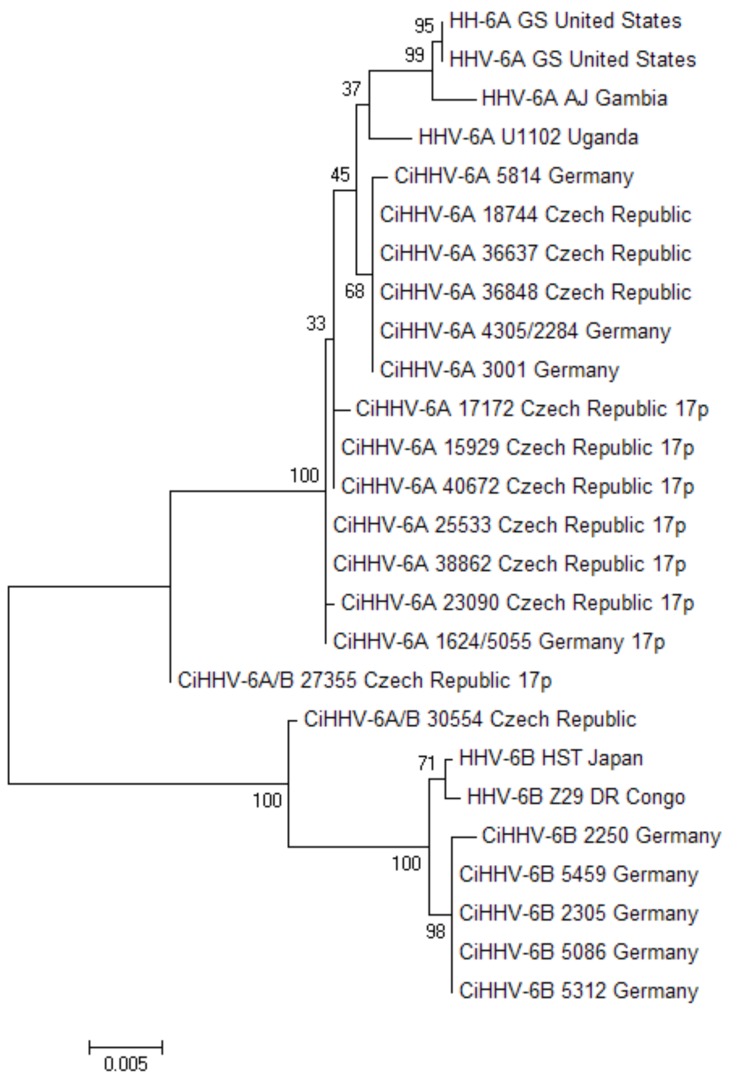
Phylogenetic relationship of coding sequences from patients with CiHHV-6A integrated at the subtelomere of human chromosome 17p. Sequences from the 4 genes were described previously from both CiHHV-6A and CiHHV-6B patients [21]. The sequences were then catenated and phylogenetic reconstructions made (see Figure 2). CiHHV-6A/B indicated intergenic recombinants previously identified [21]. Comparisons are made to sequences from known HHV-6A and HHV-6B genomes. Bootstrapping was conducted with 1000 replicates, and percentages are indicated at the nodes. Branch lengths were measured in number of substitutions per site as indicated by the scale.

The CiHHV-6A genomes which showed 17p integration sites were also linked phylogenetically from analyses of coding sequences. To show this, we catenated four coding gene sequences we had reported previously [21] available for eight of these 17p CiHHV-6A patients and 11 other patients with integration sites at other chromosomes (Figure 4) [21]. The sequences were aligned and a phylogenetic tree constructed which showed the CiHHV-6A integrated at chromosome 17p formed a clade basal to other CiHHV-6A and HHV-6A strains.

**Figure 5:**
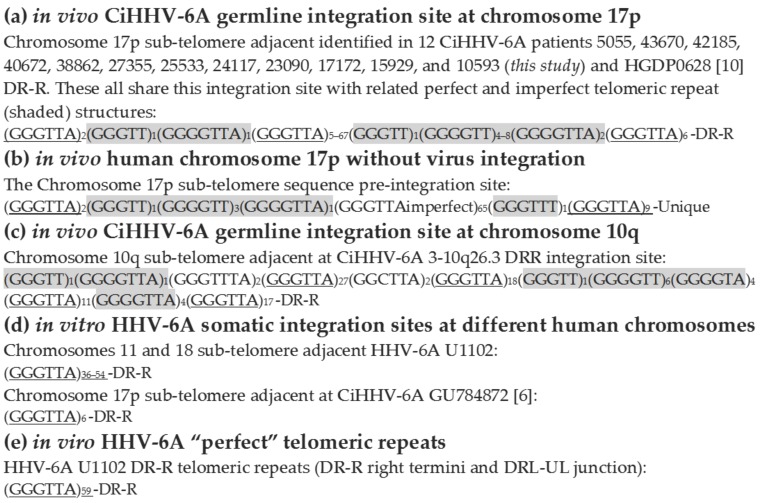
Comparison of the telomeric repeat structure between 12 CiHHV-6A integrated at chromosome 17p subtelomere and at different chromosomes 18 and 10. The repeat structures were determined and grouped into (**a**) *In vivo* virus genome integration sites at 17p telomeric repeat structure (including the 12 integration sites characterized here); (**b**) *in vivo* human 17p telomeric repeat structure at the integration site without virus genome; (**c**) *in vitro* derived human virus genome integration sites; (**d**) *in vivo* virus genome integration sites at chromosome 10q and (**e**) *in viro*, the perfect telomeric repeats in the exogenous virus towards the right termini prior to chromosomal integration. Imperfect repeats derived from the human subtelomeric region are shaded. The length differ in the number of perfect telomeric repeats in the center as shown. Comparisons are shown to two previously described integration sites at 17p with similar structure as well as to the distinct integration site in chromosome 10 and the *in vitro* integration sites at chromosome 18. Of the 12 integration sites at 17p characterized here, the largest 5055, has accession number TBA. Two previous HHV-6A 17p integration sites: HGDP0628 [10], GU784872 [6]; Chromosome 17 subtelomere, complete sequence, genbank AC240565.4; Chromosome 17 telomere adjacent genomic sequence, genbank DQ355024.2.

We next determined the telomeric repeat structure at these 17p integration sites. The virus had GGGTTA hexameric repeats like the human telomeric repeats. Since all the integration sites at 17p had identical adjacent subtelomeric unique sequence we were able to compare the CiHHV-6A integration site telomeric repeats structure to those found adjacent to the original human subtelomere region of chromosome 17, prior to virus integration. This showed that the origin of the divergent repeat structure was from this human subtelomeric region recombined at this site together with the perfect telomeric repeats from the original virus termini (Figure 5a,b,e). All share these imperfect repeats from 17p plus a terminal 6 perfect GGGTTA telomeric-like repeats prior to the adjacent DR-R sequence. The only divergence between the patients’ integration site at 17p is in the copy number of central perfect telomeric repeats which vary from 5 to 67, mean 39, median 52 (Figure 5a). This structure is distinct from the integration sites defined at the other chromosomes, 18, 10 and 11 (Figure 5c,d). Two previous integration sites characterized at 17p show a similar structure to that demonstrated here (Figure 5a). However, in one, only the signature 6 perfect telomeric repeats prior to DR are retained (Figure 5c). The *in vitro* integration site defined previously for HHV-6A U1102 also only retain the perfect telomeric repeats prior to DR (Figure 5d,e) [6]. Possibly these are *in vitro* cell culture passage effects. This overall conserved *in vivo* germline integration site structure at chromosome 17p supports a common ancestral integration event.

### 3.3. Conserved cis-Acting Signals for DNA Packaging, Replication and Gene Regulation

**Figure 6:**
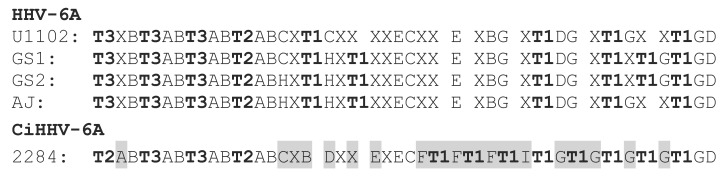
Structure of the imperfect telomeric repeat region at the UL-DRR junction. Perfect telomeric repeats are the related complement [9] in the inverse orientation in the reference genome, TAACCC, and the adjacent copy numbers are indicated as T1–T3. Imperfect telomeric repeats are represented by “**A**” for TAGGTC; “**B**” TAGCCC; “**C**” TAACCA; “**D**” TAACCG; “**E**” TAACAC; “**F**” TAACAA; “**G**” TAGCCA; “**H**” TAACTA; “**I**” TAGCAA and “X” for unrelated hexamers. Shading indicates rearranged hexamers.

To further check if the endogenous genomes were defective or competent for replication or virus reactivation, we examined *cis*-acting signals. These were compared between the CiHHV-6A integrated genomes and exogenous HHV-6A virus reference genomes. The DNA packaging motifs pac1/pac2, were retained with only a single SNP difference. The pac2 DNA packaging motif was not present at the DRR junction as shown previously [6,10]. Although the 8 kb DR region from CiHHV-6A 2284 genome showed greatest diversity, 5%, there were no major indels distinct from exogenous virus genomes, two indels of 101 and 70 bp were also present in different virus strains, with a further small 43 bp deletion in the central non-coding AT rich repetitive region observed. The imperfect telomeric/hexameric repeat region at the CiHHV-6A genome UL-DR-R junction, was similar to that in exogenous virus genomes, with 46 hexamers, with additional telomeric repeats (Figure 6).

**Figure 7:**
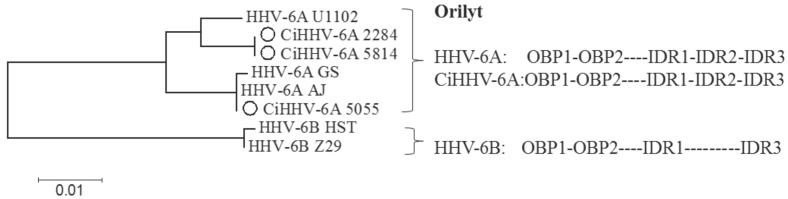
Phylogenetic analyses of the origin of lytic replication, ori-lyt in CiHHV-6A compared to HHV-6A. Replication origins from CiHHV-6A are compared to the maximally efficient ori-lyt mori region characterized by *in vitro* replication assays for HHV-6A strain U1102 [58]. HHV-6B ori-lyts are used as outgroups. The two origin binding motifs are conserved, OBP1 and OBP2 as are the indirect repeat regions, 1–3 (IDR1, IDR2, and IDR3). 100% bootstrap support for 1000 iterations at main nodes. Alignment is in Figure S2.

The origin of lytic replication, was also highly conserved, and mimicked closely either the structure of HHV-6A virus strains AJ and GS (5814, 2284/4305) in one branch, or HHV-6A U1102 (5055) in the second branch (Figure 7). This region included the 1.1 kb mori region, the minimal region of efficient replication, retaining identical origin binding protein sequences of the smallest ori, OBP1 OBP2 and only 3 SNPS in the imperfect direct repeats (IDR1, IDR2, and IDR3), regions which improve efficiency [58]. There are two small indels specific to HHV-6A U1102 retained in CiHHV-6A 5814 and 2284/4305, while CiHHV-6A 5055 includes the small deletions found in HHV-6A AJ and GS (Supplement Figure S2). Therefore, despite the repeat structure, the functional efficient origin of lytic replication is one of the most conserved regions of the genomes. Similarly, the site of CpG suppression over the immediate early genes, indicative of gene regulation by methylation, was also retained (not shown) [8]. Finally, the previously defined microRNA features are also retained in the DR [59] including the recently defined HHV-6A specific mir86 gene regulator [60]. These features all show the integrated genome retains cis-acting signals required to direct genome replication and encapsidation for virus reactivation.

## 4. Discussion

### 4.1. Gene Divergence

All 85 previously identified coding regions in HHV-6A virus genomes were identified in the CiHHV-6A genomes, with no major indels compared to the 159 kb reference. There were 16/85 genes with increased SNP density in CiHHV-6A genomes compared to HHV-6A viruses defined to date, showing up to 6% divergence. These included all the divergent genes, which had been shown to distinguish HHV-6A and HHV-6B as well as supporting their classification [27,55,56]. In contrast, an initial report on an CiHHV-6B integrated genome suggests it is almost identical to HHV-6B virus, reporting SNPs giving less than 0.2% divergence, although the genomic sequence is not published [10]. The divergent CiHHV-6A genes defined here can serve as potential new biological markers for this integrated-form and can be used to test the extant variation between CiHHV-6A and exogenous HHV-6A strains. These genes encode roles in transcriptional regulation, virus infection, cell cycle, and immune modulation; they include Roseolovirus specific functions, which can affect host tropism and immune evasion for persistence. Six genes show greater diversity in CiHHV-6A than between HHV-6A strains, *U14* (cell cycle), *U71*, *U79* (DNA replication), *U86* (IE2), *U95* (GRIM-19 mitochondria), and *U100* (infection, gQ). These highlight roles in transcriptional regulation and host tropism, the main areas for species diversification in herpesviruses. In contrast all the cis-acting signals for virus replication or DNA packaging into the virion are completely conserved. Since both gene expression and genome replication have been demonstrated, production of virus may be possible from the endogenous genomes. The data described here show preservation of the complete gene complement and *cis*-acting signals for gene regulation, replication and encapisidation. However, the genome appears divergent and therefore may result in reactivation of CiHHV-6A virus with distinct properties from known exogenous HHV-6A. Moreover, gene expression from the endogenous CiHHV-6A genome may have effects *in vivo* independent of virus production.

Comparisons of some HHV-6A and HHV-6B gene encoded functions show differences in immune adaptation and these genes are divergent in CiHHV-6A. Like other human pathogens, HHV-6B encodes a function, *IE1* (immediate early protein 1), which efficiently evades human interferon beta by disrupting signalling through the Janus Kinase/Signal Transducer and Activator of Transcription (JAK/STAT) pathway. In contrast, HHV-6A remains sensitive, a possible marker for zoonosis, the IE lacks a specific gene insertion present in HHV-6B that mediates interferon evasion and the same is seen in CiHHV-6A [61]. *U54A* is a transactivator of NFAT in contrast to *U54B* which is an inhibitor, and the motif contributing to this distinction is retained in CiHHV-6A, with further divergent sequences [53]. Moreover, HHV-6A *U20A* downregulates major histocompatibility complex, MHC, class I more efficiently than HHV-6B *U20B* showing unregulated immune surveillance, this gene is in CiHHV-6A [62]. Furthermore, HHV-6A gH/gL/gQ1/gQ2 complex mediates higher affinity virus interactions with CD46 present on almost all cells, which could disrupt immune signalling, whereas the HHV-6B component is restricted [63,64,65]. All these gene functions are also part of the gene set divergent in the CiHHV-6A genomes characterized here and their phylogenetic analyses show they are basal to related known exogenous HHV-6A genes. This would support the hypothesis that HHV-6A strains are derived from reactivated endogenous CiHHV-6A genomes, or that reactivated CiHHV-6A could produce a distinct emergent infection.

### 4.2. Integration Site

The central European CiHHV-6A groups, shown by the Czech and German patients analyzed here, were dominated by integration at the chromosome 17p subtelomere. Since integration at the 17p telomere appears more frequently, it may be ancestral, particularly for this region. Although, one other reported integration at 17p, which has also derived sequence from the integration site, has related DR sequences and is from a donor from Mideast Asia (Figure 3) [10]. Human telomeres that carry CiHHV-6A/B genome integrations have been shown to be shorter and chromosome 17 itself is shorter possibly explaining its prevalence as an integration site [10,66].

The shared structure of the 17p integration site and phylogenetically related coding sequences from the integrated genomes, indicate a related pedigree and a rare event spread now in a Mendellian mechanism. The ancestral node for sequence integrated at 17p appears basal to other CiHHV-6A or exogenous HHV-6A, and further characterisation of additional strains could resolve the ancestral nature of this integration site. All twelve of the 17p integrations sites characterized, shared imperfect repeats structures, and only differed in the copy number of perfect telomeric repeats. This telomeric repeat copy variation was shown for two related cases with CiHHV-6B integration at the Xp chromosome [11] and also in exogenous HHV-6A strains from different individuals [67]. Analyses of the repeat structures, showed the imperfect repeats were derived from the chromosome 17p subtelomere. The 17p integration site is distinct from that in other chromosomes suggesting multiple ancestral lineages. Alternatively, integration at 17p could be ancestral for CiHHV-6A, with other chromosomal integrations resulting from DNA replication and reintegration within the nucleus.

The European CiHHV-6A 17p group also have fixed a gene with a frameshift homopolymeric region (FHR) [21]. FHR are a recently recognized common mechanism in herpesvirus genomes, generating further diversity [68]. In CiHHV-6, unlike many defective endogenous viruses, we showed the gene fixed is an active version, encoding a potent chemokine, rarely shown in exogenous virus [21], a potential factor or biomarker in linked inflammatory disease patients.

### 4.3. Prevalence and Emergence

The prevalence of CiHHV-6A in continental Europe as shown in the meta-analysis may point to early germline integration of the virus genomes and spread in populations emigrating from Africa. This is consistent with the host co-evolutionary hypothesis for herpesvirus [46,69,70] which would place HHV-6A and HHV-6B divergence after common ancestors represented at nodes distinct to related chimpanzee HHV-6-like roseolovirus (see gb AY359407 and gb AY854171) as described [71]. Following the co-evolution model this would also place the ancestral node for HHV-6A and HHV-6B after the divergence of humans from chimpanzees (between two and six million years ago). Since CiHHV-6A is more divergent and has a phylogenetic relationship basal to exogenous HHV-6A and B or CiHHV-6B, this raises the possibility of endogenous CiHHV-6A derived from infections in a hominid ancestor. Computational analyses of exact timelines may be confounded by differing host and virus mutation rates, distinct selective pressures upon integration, recombination, as well as varying multiple integration/excision cycles and are under further investigation. Moreover, previous coevolutionary analyses suggested HHV-6A/B may be zoonoses from ungulates [72] and partial genomic sequences related to HHV-6A/B were identified integrated in a Tarsier genome also supporting early origins, but this was markedly degenerate with defective, interrupted genes, in complete contrast to the intact CiHHV-6A genome described here, and likely an independent or isolated integration event [73].

To date, the prevalence of CiHHV-6A and CiHHV-6B are overall similar at 0.2 and 0.4 in combined studies screening over 19,000 individuals, both donors and patients from different global regions (Table S1). There is increased prevalence of CiHHV-6A in Europe as shown here and decreased in Japan, 0.3% compared to 0.04%, while it has not yet been identified in Southern Africa (<0.1%) and only one case in Northern Africa. In contrast, exogenous HHV-6A is a circulating virus in Southern Africa commonly detected in asymptomatic infants, and occasionally in symptomatic groups, with extensive characterisation at different loci in multiple clinical strains and two complete genomes [8,21,23,49,74,75] (Table 1). While in Europe, infant HHV-6A infections are rarely detected. Therefore, known exogenous HHV-6A virus could be earlier reactivations from CiHHV-6A, and the current proportions of virus and integrated genomes affecting European populations a consequence of migration and population bottlenecks combining both Mendellian inheritance and patterns of infectious spread.

Both CiHHV-6A and HHV-6A have been associated with inflammatory cardiovascular disease, myocarditis and idiopathic-cardiomyopathy [18,44,76,77]. Recent Canadian cohort analyses also show CiHHV-6A/B linked with angina [20]. The genomic analyses presented here was on European CiHHV-6A patients with recurrent heart disease [18], where heart failure symptoms correlated with gene expression, and anti-viral treatment removed both symptoms and gene expression. In the CiHHV-6A patients, virus proteins and particles were detected in the degenerating myocytes [18]. We showed two of these CiHHV-6A cardiac patients had superinfecting exogenous HHV-6A virus, and previous studies detected gene expression from the endogenous integrated-genome. [21]. Our analyses of this complete CiHHV-6A genome demonstrated it has potential for virus reactivation. The gene divergence in the CiHHV-6A genomes was distinct from that of minor variants of exogenous HHV-6A identified in the same individuals by deep sequencing. These exogenous HHV-6A SNPs conformed to known reference HHV-6A SNPs indicating circulating HHV-6A in this region is related to known virus. This raises the possibility that superinfecting exogenous virus induces symptoms through reactivation of gene expression, DNA replication or virus from the distinct endogenous integrated-genomes. Microbial superinfection can reactivate genome replication *in vitro* [78] and this also appears sporadically [10,21,78]. Actual contemporary transmission of reactivated CiHHV-6A encapsidated, infectious virus needs further study, but the genomic characterisations here show they have all necessary components to make virus and provide the genetic tools to investigate further.

Although limited by the number of exogenous virus genomes available to date, this study is the first to our knowledge to describe the CiHHV-6A integrated genome, showing it is fully intact, and therefore has the capacity for virus reactivation. It also establishes the relationship to known exogenous HHV-6A virus, provides instruments to test relationships and analyze linked pathology, previously confounded by these close but distinct genetic forms.

## Supplementary Materials

The following are available online at www.mdpi.com/1999-4915/8/1/19/s1, Table S1: Geographic prevalence studies separated into CiHHV-6A and CiHHV-6B; Table S2: HHV-6A SNPs detected in CiHHV-6A by deep sequencing; Figure S1: Structure of CiHHV6A/B and HHV-6A/B DR; Figure S2: The origin of lytic replication, mori, of ciHHV-6A 5055/1624, 5814, and 2284/4305 compared to virus reference HHV-6A U1102, AJ, GS and HHV-6B Z29, HST.
