# Supplementary material for: Complete Genome Sequence of Germline Chromosomally Integrated Human Herpesvirus 6A and Analyses Integration Sites Define a New Human Endogenous Virus with Potential to Reactivate as an Emerging Infection
Source: Viruses. 2016 Jan 15;8(1):19. doi: 10.3390/v8010019 (PMC4728579; doi:10.3390/v8010019)
Supplement: Supplementary file 1 [file viruses-08-00019-s001.pdf]

Supplementary Materials: Complete Genome Sequence of Germline Chromosomally Integrated Human Herpesvirus 6A and Analyses Integration Sites Define a New Human Endogenous Virus with Potential to Reactivate as an Emerging Infection

Joshua Tweedy, Maria Alexandra Spyrou, Max Pearson, Dirk Lassner, Uwe Kuhl and Ursula A. Gompels

Table S1. Geographic prevalence studies separated into CiHHV-6A and CiHHV-6B.

Study	Country	*n*	CiHHV-6A (%)	CiHHV-6B (%)	References
			**Donors**		
Cord blood	USA (NY)	5638	[19 *] 12 (0.2)	[38 *] 25 (0.4)	(Hall *et al.*, 2008, 2004)
Blood donor-adult	USA (Texas)	100	0 (<1.0)	1 (1.0)	(Hudnall *et al.*, 2008)
Blood donor	Canada (Ontario)	288	0 (<0.3)	0 (<0.3)	(Gravel *et al.*, 2013)
Blood donors-adult	France	200	0 (<0.5)	1 (0.5)	(Geraudie *et al.*, 2012)
Blood donor–adult	UK (London)	500	0 (<0.2)	4 (0.8)	(Leong *et al.*, 2007)
Serum bank-child	UK	610	4 + (0.7)	6 + (1.0)	(Ward *et al.*, 2005)
Blood controls	UK (Northern)	563	1 (0.2)	10 (1.7)	(Bell *et al.*, 2014) ^
Nails adult	Czech Republic	421	1 (0.2)	3 (0.7)	(Hubacek *et al.*, 2013)
Normal birth/infant-saliva, sera, nails	Southern Africa (Zambia)	495	0 (<0.2)	0 (<0.2)	#
Region Totals	North America N	6026	12 (0.2)	26 (0.4)	
	Europe E	2582	6 (0.2)	24 (0.9)	
Donor Total	NA + E	8608	18 (0.2)	50 (0.6)	
			**Patients**		
SOT-Liver	USA	548	1 (0.2)	6 (1.1)	(Lee *et al.*, 2012)
SOT-Kidney	USA	46	0 (<2.0)	1 (2.2)	(Lee *et al.*, 2011)
Leukemic children	Canada (Ontario)	287	1 (0.3)	0 (<0.3)	(Gravel *et al.*, 2013)
SOT-mixed. Blood, tissue, hair samples	Italy	135	1 (0.7)	0 (<0.8)	(Potenza *et al.*, 2009)
SCT-Blood, hair samples	Italy	70	0 (<1.4)	1 (1.4)	(Potenza *et al.*, 2009)
CSF-encephalitis referral child/adult	UK	522	1 (0.2)	5 (1.0)	(Ward *et al.*, 2007)
Hodgkins lymphoma	UK (Northern)	936	1 (0.1)	15 (1.6)	(Bell *et al.*, 2014) ^
Cardiac referrals	Germany	3610	7 (0.2)	13 (0.4)	(Tweedy *et al.*, 2015)
Malignant disease-blood	Czech Republic	812	7 (0.9)	2 (0.2)	(Hubacek *et al.*, 2013)
Leukemia-child-blood	Czech Republic	339	4 (1.2)	1 (0.3)	(Hubacek *et al.*, 2009)
Transplant donors/recipients-blood, herpesvirus referrals	Japan (Osaka)	2332	1 (0.04)	4 (0.2)	(Tanaka-Taya *et al.*, 2004)
Neonatal intensive care unit, sera	Southern Africa (Zambia)	303	0 (<0.3)	0 (<0.3)	(Tembo *et al.*, 2014) ^
Leukemia-blood	North Africa (Tunisia)	73	0 (<1%)	1 (1.2%)	(Faten *et al.*, 2012)
Region Totals	North America	881	2 (0.2)	7 (0.8)	
	Europe	6424	21 (0.3)	37 (0.6)	
	Japan, J	2332	1 (0.04)	4 (0.2)	
Patient Total	NA + E + J + A	9719	23 (0.3)	34 (0.4)	

Table S1. *Cont.*

Study	Country	*n*	CiHHV-6A (%)	CiHHV-6B (%)	References
		**Donor + Patients**			
Region Totals	North America	6907	14 (0.2)	33 (0.5)	
	Europe	9006	27 (0.3)	61 (0.7)	
	Japan	2332	1 (0.04)	4 (0.2)	
	Africa, A	868	0 (<0.1)	1 (0.1)	
**ALL**	**NA + E + J + A**	**19,113**	**41 (0.2)**	**74 (0.4)**	

* Original numbers screened, then those positively identified listed next; +4 and 6 positively identified; SOT—solid organ transplantation recipients; CSF—cerebral spinal fluid; NA North America; # Musonda, K. and Gompels, U.A., Analyses congenital infections with betaherpesviruses in Zambia, unpublished; manuscript in preparation; ^ References [1,15] type by polymerase gene, which HHV-6A variation may confound [4,16].

Table S2. HHV-6A SNPs detected in CiHHV-6A by deep sequencing.

No.	SNPs U54 HHV-6A > CiHHV-6A	SNPs U54 Amino Acid	HHV-6A U1102	% HHV-6A Minor Variant SNPs in CiHHV-6A		
	**HHV-6A:U1102, GS & AJ**	**Changes**	**Position**	**2284**	**5055**	**5814**
1	A > G	-	86,106	<	<	<
2	A > G	-	86,142	17	<	<
3	G > A (AJ only)	-	86,195	17	<	<
4	A > G (AJ only)	I > T	86,329	16	<	<
5	G > T (AJ only)	L > I	86,372	16	<	<
6	T > C	[S, A, T > M]	86,379	17	<	<
7	G > A	[S, A, T > M]	86,380	18	<	<
8	C (U1102) A (GS) > T	S, A, T > M	86,381	<	<	<
9	T > C	T > A	86,387	16	<	<
10	G > C	-	86,400	17	<	<
11	T > C (GS only)	-	86,514	14	<	<
12	T > C	T > A	86,537	30	<	4
13	G > T	T > N	86,608	31	<	<
14	A > G	-	86,613	31	<	<
15	A > G	-	86,619	31	<	<
16	C > A	V > F	86,627	30	<	<
17	G > A	-	86,638	28	<	<
18	G > A	P > S	86,645	25	<	<
19	A > G	-	86,720	17	<	<
20	T > A (GS only)	-	86,724	<	<	<
21	G > T	A > D	86,761	16	<	<
22	A > C	[I, L > R]	86,791	17	<	<
23	T > G (GS only)	I, L > R	86,792	<	<	<
24	T > C (AJ only)	N > D	86,801	17	<	<
25	G > T (U1102)	K, N > I	86,811	<	<	<
26	T > A	[K, N > I]	86,812	16	<	<
27	A > G	-	86,859	14	<	<
28	T > C	N > D	86,891	19	<	<
29	C > G (U1102 only)	G > A	86,893	<	<	<
30	G > T	T > N	86,896	19	<	<
31	C > T (U1102 only)	-	86,943	<	<	<
32	A > C (GS only)	N > K	86,949	<	<	<
33	G > T (U1102 only)	Q > K	87,011	<	<	<
34	T > C	K > R	87,016	22	<	<
35	A > G	-	87,036	21	<	<
36	C > T	A > T	87,092	16	<	<
37	C > T (U1102 and AJ)	R > H	87,100	16	<	<
38	A > G (GS only)	S > P	87,110	<	<	<

Table S1. *Cont.*

**No.**	**SNPs U54 HHV-6A > CiHHV-6A**	**SNPs U54 Amino Acid**	**HHV-6A U1102**	**% HHV-6A Minor Variant SNPs in CiHHV-6A**		
	**HHV-6A:U1102, GS & AJ**	**Changes**	**Position**	**2284**	**5055**	**5814**
39	C > A (GS only)	A > S	87,119	<	<	<
40	T > C (GS only)	M > V	87,128	<	<	<
41	G > C	N > K	87,129	20	<	<
42	G > A	H > Y	87,137	19	<	<
43	T > G	R > S	87,171	18	<	<
44	A > T	I > N	87,199	18	<	2
45	C > A	Q > R	87,204	18	<	<
46	T > C	Q > R	87,205	18	<	3
47	T > C	T > A	87,266	15	<	<
48	A > G	I > T	87,289	15	<	<
49	T > C (GS)	-	87,299	<	<	<
50	A > G (U1102 and AJ)	S > P	87,308	15	<	<
51	A > G (GS)	S > P	87,314	<	<	<

Mean read depths from new sequences from this study (methods 2.3) were for endogenous CiHHV-6A genomes from patients 2284, 5055 and 5814 it was 314, 9943 and 8941 respectively. Comparisons were made to all available reference genomes, from exogenous HHV-6A strains U1102, GS and AJ (methods 2.2). SNP% cutoffs were <0.5% reads indicated by <. Coding from the opposite strand. Brackets [ ] indicate same codon giving the coding change.

a. telomere - DR-L-------------UL------------DR-R-sub-telomere-----//----centromere
CiHHV-6A/B

b. DR: pac1-T1-DR1-DR6-T2-pac2

Figure S1. (**a**) Structure of CiHHV-6A/B underlined as integrated into the sub-telomeric region of human chromosomes. DR-L is the left direct repeat and DR-R is the right direct repeat in the prototype orientation of the virus genome, and bound the unique region, U, encoding most coding sequences; (**b**) The structure of the DR region from HHV-6A/B which includes the pac 1 and pac 2 DNA packaging signals, imperfect telomeric repeat region T1, perfect telomeric repeat region T2, and spliced coding sequences for genes DR1 and DR6. In the CiHHV-6A/B genomes the DR regions do not have the terminal pac sites.

```
                                 Start of U41  Start ori-lyt(mori)67610
HHV-6A.U1102    AACGGGAGCAGAAACTACCGTTTCGTTTTCATCAGCCATCTTTGTGGATTTGATCACAGA 67641
GS              AACGGGAGCAGAAACTACCGTTTCGTTTTCATCAGCCATCTTTGTGGATTTGATCACAAA
AJ              AACGGGAGCAGAAACTACCGTTTCGTTTTCATCAGCCATCTTTGTGGATTTGATCACAAA
CiHHV-6A.5055   AACGGGAGCAGAAACTACCGTTTCGTTTTCATCAGCCATCTTTGTGGATTTGATCACAAA
2284            AACGGGAGCAGAAACTACCGTTTCGTTTTCATCAGCCATCTTTGTGGATTTGATCACAAA
5814            AACGGGAGCAGAAACTACCGTTTCGTTTTCATCAGCCATCTTTGTGGATTTGATCACAAA
HHV-6B.Z29      AACAGGAGCAGAAACCACCGTTTCGTTTTCATCAGCCATCTTTGTGGATTCGATCACAAA
HST             AACAGGAGCAGAAACCACCGTTTCGTTTTCATCAGCCATCTTTGTGGATTCGATCACAAA
                *** *********** ********************************** ******* *

HHV-6A.U1102    AACAGAGATAATGGGGTTTTGTGGTGAAATCCTTATATATTATGTTTGACGTAACATAAC
GS              AACAAAGATAATGGGGTTTTGTGGTGAAATCCTTATATATTATGTTTGACGTAACATAAC
AJ              AACAAAGATAATGGGGTTTTGTGGTGAAATCCTTATATATTATGTTTGACGTAACATAAC
CiHHV-6A.5055   AACAAAGATAATGGGGTTTTGTGGTGAAATCCTTATATATTATGTTTGACGTAACATAAC
2284            AACAAAGATAATGGGGTTTTGTGGTGAAATCCTTATATATTATGTTTGACGTAACATAAC
5814            AACAAAGATAATGGGGTTTTGTGGTGAAATCCTTATATATTATGTTTGACGTAACATAAC
HHV-6B.Z29      AACAAAGATAATGGGTTTTTGTGGTGAAATCCTTATATATTATGTTTGACGTAACATAAC
HST             AACAAAGATAATGGGTTTTTGTGGTGAAATCCTTATATATTATGTTTGACGTAACATAAC
                **** ********** ********************************************

HHV-6A.U1102    ACGCGTCATCAAACATAAGAGTAAACCACAAGTTGAATTATACCGTTTTCTATATGAGGT
AJ              ACGCGTCATCAAACATAAGAGTAAACCACAAGTTGGATTATACCGTTTTCTATATGAGGT
GS              ACGCGTCATCAAACATAAGAGTAAACCACAAGTTGGATTATACCGTTTTCTATATGAGGT
CiHHV-6A.5055   ACGCGTCATCAAACATAAGAGTAAACCACAAGTTGGATTATACCGTTTTCTATATGAGGT
2284            ACGCGTCATCAAACATAAGAGTAAACCACAAGTTGAATTATACCGTTTTCTATATGAGGT
5814            ACGCGTCATCAAACATAAGAGTAAACCACAAGTTGAATTATACCGTTTTCTATATGAGGT
HHV-6B.Z29      ACGCGTCATCAAACATAAAAGTAAACCACAAGTTGAAATATACCGTTTTCTATATGAGTT
HST             ACGCGTCATCAAACATAAAAGTAAACCACAAGTTGAAATATACCGTTTTCTATATGAGTT
                ****************** **************** * ******************** *

HHV-6A.U1102    TTACGGTCAAAAGAAAAACGATTTTTTTATGCAAATATTTTCCACGCAGATGATATGACA
GS              TTACGGACAAAAGAAAAACGATTTTTTTATGCAAATATTTTCCACGCAGATGATATGACA
AJ              TTACGGACAAAAGAAAAACGATTTTTTTATGCAAATATTTTCCACGCAGATGATATGACA
CiHHV-6A.5055   TTACGGACAAAAGAAAAACGATTTTTTTATGCAAATATTTTCCACGCAGATGATATGACA
2284            TTACGGACAAAAGAAAAACGATTTTTTTATGCAAAGATTTTCCACGCAGATGATATGACA
5814            TTACGGACAAAAGAAAAACGATTTTTTTATGCAAAGATTTTCCACGCAGATGATATGACA
HHV-6B.Z29      TTACGGACAAAAGAAAAACGATTTTCTTATGCAAATATTTTCCACGCAGATGACATGACA
HST             TTACGGACAAAAGAAAAACGATTTTCTTATGCAAATATTTTCCACGCAGATGACATGACA
                ****** ****************** ********* ***************** ******
                                                    OBP2
HHV-6A.U1102    CGCCCCTTAATTTAAATTTATGCAAATCGTCGTCCACCTCAGGTACAATAGTATATTATA 67822
GS              CGCCCCTTAATTTAAATTTATGCAAATCGTCGTCCACCCCAGGTACAATAGTATATTATA
AJ              CGCCCCTTAATTTAAATTTATGCAAATCGTCGTCCACCTCAGGTACAATAGTATATTATA
CiHHV-6A.5055   CGCCCCTTAATTTAAATTTATGCAAATCGTCGTCCACCTCAGGTACAATAGTATATTATA
2284            CGCCCCTTAATTTAAATTTATGCAAATCGTCGTCCACCTCAGGTACAATAGTATATTATA
5814            CGCCCCTTAATTTAAATTTATGCAAATCGTCGTCCACCTCAGGTACAATAGTATATTATA
HHV-6B.Z29      CGCCCCTTAATTTAAATTTATGTAAATCGTCGTCCACCTCAGGTACAATAGTATATTATA
HST             CGCCCCTTAATTTAAATTTATGTAAATCGTCGTCCACCTCAGGTACAATAGTATATTATA
                ********************** *************************************
                                                 OBP1
HHV-6A.U1102    TATATATTATTTTAATAAACTTATTGAGGACGGGAGAACGAGGGCGTGGCGTTTACGTCA 67882
GS              TATATATTATTTTAATAAACTTATTGAGGACGGGAGAACGAGGGCGTGGCGTTTACGTCA
AJ              TATATATTATTTTAATAAACTTATTGAGGACGGGAGAACGAGGGCGTGGCGTTTACGTCA
CiHHV-6A.5055   TATATATTATTTTAATAAACTTATTGAGGACGGGAGAACGAGGGCGTGGCGTTTACGTCA
2284            TATATATTATTTTAATAAACTTATTGAGGACGGGAGAACGAGGGCGTGGCGTTTACGTCA
5814            TATATATTATTTTAATAAACTTATTGAGGACGGGAGAACGAGGGCGTGGCGTTTACGTCA
HHV-6B.Z29      TATATAGTTTTTTAATAAACTTATTGAGGACGGGAGAACGAAGGCGTGGCGTTTACGTCA
HST             TATATAGTTTTTTAATAAACTTATTGAGGACGGGAGAACGAAGGCGTGGCGTTTACGTCA
                ****** * ******************************** ******************

HHV-6A.U1102    TAGCCTAATTATGCATTCTCAGAACAGGATTTAAGAAGGCTGCGAGCGGCCGACTGTTCA
GS              TAGTCTAATTATGCATTCTCAGAACAGGATTTAAAAAGGCTGCGAGCGGCTGACTGTTCA
AJ              TAGTCTAATTATGCATTCTCAGAACAGGATTTAAAAAGGCTGCGAGCGGCTGACTGTTCA
CiHHV-6A.5055   TAGTCTAATTATGCATTCTCAGAACAGGATTTAAAAAGGCTGCGAGCGGCTGACTGTTCA
2284            TAGCCTAATTATGCATTCTTAGAACAGGATTTAAAAAGGCTGCGAGCGGCCGACTGTTCA
5814            TAGCCTAATTATGCATTCTTAGAACAGGATTTAAAAAGGCTGCGAGCGGCCGACTGTTCA
HHV-6B.Z29      TAGCCTAATTATACATTCTCAGAACAGGATTTAAAAAGGCTGCGAGCGGCCGGCTGTTCA
HST             TAGCCTAATTATACATTCTCAGAACAGGATTTAAAAAGGCTGCGAGCGGCCGGCTGTTCA
                *** ******** ****** ************** *************** * *******

HHV-6A.U1102    GAGGGACGCTGGGGTACGGCTTGATACGTTTGATTGAAAATGATTCTTCGTGTGCTATTT
GS              GAGGGACGCTGGGGTACGGCTTGAGACGTTTGATTGAAAATGATTCTTCGTGTGCTATTT
AJ              GAGGGACGCTGGGGTACGGCTTGAGACGTTTGATTGAAAATGATTCTTCGTGTGCTATTT
CiHHV-6A.5055   GAGGGACGCTGGGGTACGGCTTGAGACGTTTGATTGAAAATGATTCTTCGTGTGCTATTT
2284            GAGGGACGCTGGGGTACGGCTTGAGACGTTTGATTGAAAATGATTCTTCGTGTGCTATTT
5814            GAGGGACGCTGGGGTACGGCTTGAGACGTTTGATTGAAAATGATTCTTCGTGTGCTATTT
```

```
HHV-6B.Z29      GAGGGACGCTGGGGTACGACTTGAGACGTTTGACTGAAAATGATCCTTCGTGTACTATTT
HST             GAGGGACGCTGGGGTACGACTTGAGACGTTTGACTGAAAATGATCCTTCGTGTACTATTT
                ****************** ***** ******** ********** ******** ******

HHV-6A.U1102    TCTGCAAAAAAATTAATTCGCCGGCGACAGTAAACTTTTCAGCGGAATTTCAAAAATTAA
GS              TCTGCAAAAAAATTAATTCGCCGGCGACAGTAAACTTTTCAGCGGAATTTCAAAAATTAA
AJ              TCTGCAAAAAAATTAATTCGCCGGCGACAGTAAACTTTTCAGCGGAATTTCAAAAATTAA
CiHHV-6A.5055   TCTGCAAAAAAATTAATTCGCCGGCGACAGTAAACTTTTCAGCGGAATTTCAAAAATTAA
2284            TCTGCAAAAAAATTAATTCGCCGGCGACAGTAAACTTTTCAGCGGAATTTCAAAAATTAA
5814            TCTGCAAAAAAATTAATTCGCCGGCGACAGTAAACTTTTCAGCGGAATTTCAAAAATTAA
HHV-6B.Z29      TCTACAAAAAAATTAATTTGCCGGCGACAGTAAACTTTTCAGCGGAATTTCAAAAATTAA
HST             TCTACAAAAAAATTAATTTGCCGGCGACAGTAAACTTTTCAGCGGAATTTCAAAAATTAA
                *** ************** *****************************************
                                        IDR1
HHV-6.U1102     TTCCATATGTAATTTAAGCATTTTAAAACGTATAACTCACAACGTGAAAACATATTTACG 68112
GS              TTCCACATGTAATTTAAGCATTTTAAAACGTATAACTCACAACGTGAAAACATATTTACG
AJ              TTCCACATGTAATTTAAGCATTTTAAAACGTATAACTCACAACGTGAAAACATATTTACG
CiHHV-6A.5055   TTCCACATGTAATTTAAGCATTTTAAAACGTATAACTCACAACGTGAAAACATATTTACG
2284            TTCCATATGTAATTTAAGCATTTTAAAACGTATAACTCACAACGTGAAAACATATTTACG
5814            TTCCATATGTAATTTAAGCATTTTAAAACGTATAACTCACAACGTGAAAACATATTTACG
HHV-6B.Z29      TTCCACATGTAATTTAAGCATTTTAAAACGTATAACTCACAAGGTGAAAACATATTTACG
HST             TTCCACATGTAATTTAAGCATTTTAAAACGTATAACTCACAAGGTGAAAACATATTTACG
                ***** ************************************ *****************

HHV-6A.U1102    AATACAGTAGTTTTCGTGATATTTTT-CGAAATTAAAAAATTTTAAATCGGGTAAATGAT
GS              AATACAGTAGTTTCCGTGATATTTTTTCGAAATTAAAAAATTTTAAATCGGGTAAATGAT
AJ              AATACAGTAGTTTCCGTGATATTTTTTCGAAATTAAAAAATTTTAAATCGGGTAAATGAT
CiHHV-6A.5055   AATACAGTAGTTTCCGTGATATTTTTTCGAAATTAAAAAATTTTAAATCGGGTAAATGAT
2284            AATACAGTAGTTTTCGTGATATTTTTTCGAAATTAAAAAATTTTAAATTGGGTAAATGAT
5814            AATACAGTAGTTTTCGTGATATTTTTTCGAAATTAAAAAATTTTAAATTGGGTAAATGAT
HHV-6B.Z29      AATACAGTAGTTTTCGTGATATTTTTTAAAAATTAATAAATTTTAAATCGTGTAAGTAAT
HST             AATACAGTAGTTTTCGTGATATTTTTTAAAAATTAATAAATTTTAAATCGTGTAAGTAAT
                ************* ************   ******* *********** * **** * **
                            U1102 indel-1
HHV-6.U1102     AAAGCATATTATTAGATTCTTCACGTTAACAAAGCAAGTTTTTTTGAGGTTTTCGGTATA 68242
GS              AAAGCATACTA-------------------------AGTTTTTT-GAGGTTTTCGGTATA
AJ              AAAGCATACTA-------------------------AGTTTTTT-GAGGTTTTCGGTATA
CiHHV-6A.5055   AAAGCATACTA-------------------------AGTTTTTT-GAGGTTTTCGGTATA
2284            AAAGCATATTATTAGATTCTTCACGTTAACAAAGCAAGTTTTTT-GAGGTTTTCGGTATA
5814            AAAGCATATTATTAGATTCTTCACGTTAACAAAGCAAGTTTTTT-GAGGTTTTCGGTATA
HHV-6B.Z29      AAAGCATACTATTAGATTCTTCACGTTAACAGAGCAAGCTCTTC-GAGGTTTTCAGTAGA
HST             AAAGCATACTATTAGATTCTTCACGTTAACAGAGCAAGCTCTTC-GAGGTTTTCAGTAGA
                ******** **                         ** * **  ********* *** *
                                                    IDR2
HHV-6A.U1102    ATTTTAACATTTACTTCCACATGTAATTTAAGCATTTTAAAACGTATAACTCACAACGTG 68302
GS              ATGTTAACATTTAGTTCCACATGTAATTTAAGCGTTTTAAAACGTATAGCTCACAACGTG
AJ              ATGTTAACATTTAGTTCCACATGTAATTTAAGCGTTTTAAAACGTATAGGTCACAACGTG
CiHHV-6A.5055   ATGTTAACATTTAGTTCCACATGTAATTTAAGCGTTTTAAAACGTATAGCTCACAACGTG
2284            ATTTTAACATTTAGTTCCACATGTAATTTAAGCATTTAAAAACGTATAACTCACAACGTG
5814            ATTTTAACATTTAGTTCCACATGTAATTTAAGCATTTAAAAACGTATAACTCACAACGTG
HHV-6B.Z29      ATTTCAATATTTA-----------------------------------------------
HST             ATTTCAATATTTA-----------------------------------------------
                ** * ** *****

HHV-6A.U1102    AAAACATTTACGAATACAGTAGTTTTAGTGATGTTTTTTCGAAATTCAAAAAGTTTAAAT
GS              AAAACATTTACGAATACAGTAGTTTTCGTGATATTTTTTCGAAATTCAAAAATTTTAAGT
AJ              AAAACATTTACGAATACAGTAGTTTTCGTGATATTTTTTCGAAATTCAAAAATTTTAAAT
CiHHV-6A.5055   AAAACATTTACGAATACAGTAGTTTTCGTGATATTTTTTCGAAATTAAAAAATTTTAAAT
2284            AAAACATTTACGAATACAGTAGTTTTCGTGATATTTTTTCGAAATTCAAAAAGTTTAAAT
5814            AAAACATTTACGAATACAGTAGTTTTCGTGATATTTTTTCGAAATTCAAAAAGTTTAAAT
HHV-6B.Z29      ------------------------------------------------------------
HST             ------------------------------------------------------------
                                                                IDR3
HHV-6A.U1102    CGGGTAAATGATAAAGCATATTATTAGATTACACATGTAATTTAAGCATTTTAAAACGTA 68422
GS              CGGGTAAATGATAAAGCATATTATTAGATTCCACATGTAATTTAAGCATTTTAAAACGTA
AJ              CGGGTAAATGATAAAGCATATTATTAGATTCCACATGTAATTTAAGCATTTTAAAACGTA
CiHHV-6A.5055   CGGGTAAATGATAAAGCATATTATTAGATTCCACATGTAATTTAAGCATTTTAAAACGTA
2284            CGGGTAAATGATAAAGCATGTTATTAGATTACACATGTAATTTAAGCATTTTAAAACGTA
5814            CGGGTAAATGATAAAGCATGTTATTAGATTACACATGTAATTTAAGCATTTTAAAACGTA
HHV-6B.Z29      ---------------------------ATTCCACATGTAATTTAAGCATTTTAAAATGTA
HST             ---------------------------ATTCCACATGTAATTTAAGCATTTTAAAATGTA
                                           *** ************************* ***

HHV-6A.U1102    GAATTCACAAAGTGACAAAACATT---AAATACAGTAGTTTTCACGGTATTTTTTGAAAT
GS              GAATTCACAAAGTGACAAAACATTCACAAATACAGTAGTTTTCACGGTATTTTTCGGGAT
AJ              GAATTCACAAAGTGACAAAACATTCACAAATACAGTAGTTTTCACGGTATTTTTCGGGAT
CiHHV-6A.5055   GAATTCACAAAGTGACAAAACATTCACAAATACAGTAGTTTTCACGGTATTTTTCGGGAT
```

```
2284            GAATTCACAAAGTGACAAAACATTGACGAATACAGTAGTTTTCACGGTATTTTTTGGAAT
5814            GAATTCACAAAGTGACAAAACATTGACGAATACAGTAGTTTTCACGGTATTTTTTGGAAT
HHV-6B.Z29      GAATTCACAAAGTGACAAAACATTCACAAATACAGTAGTTTTCACGGTATTTTT-GAAAT
HST             GAATTCACAAAGTGACAAAACATTCACAAATACAGTAGTTTTCACGGTATTTTT-GAAAT
                ************************    *************************** * **


HHV-6A.U1102    TAA---ATTTTAAATCGGGTACATGATAAAGCATGCTGTTAAATTCACGTTTTTAGGGCA
GS              TAA---ATTTTAAATCGGGTAAATGACAAAGCATGCTGTTAAATTCACGTTTTTAGGGCA
AJ              TAA---ATTTTAAATCGGGTAAATGACAAAGCATGCTGTTAAATTCACGTTTTTAGGGCA
CiHHV-6A.5055   TAA---ATTTTAAATCGGGTAAATGACAAAGCATGCTGTTAAATTCACGTTTTTAGGGCA
2284            TAA---ATTTTAAATTGGGTAAATGATAAAGCATGCTGTTAAATTCACATTTTTAGGGCA
5814            TAA---ATTTTAAATTGGGTAAATGATAAAGCATGCTGTTAAATTCACATTTTTAGGGCA
HHV-6B.Z29      TAATAAATTTTAAATCGGGTAAATGGTAAAGCATGCTGTTAAATTCACGTTAACAGAGCA
HST             TAATAAATTTTAAATCGGGTAAATGGTAAAGCATGCTGTTAAATTCACGTTAAAAGAGCA
                ***   ********* ***** ***  ********************* **   ** ***

HHV-6A.U1102    AGTTCTGCGAGGTTTGCTTTGTGATTATCAGTGAATGTATTCAGCGTTTCAATTTTAGAT
GS              AGTTCTTCGAGGTTTGCTCTGTGATTATCAGTGAAT--ATTCAGCGTTTCAATTTTAGAT
AJ              AGTTCTTCGAGGTTTGCTCTGTGATTATCAGTGAAT--ATTCAGCGTTTCAATTTTAGAT
CiHHV-6A.5055   AGTTCTTCGAGGTTTGCTCTGTGATTATCAGTGAAT--ATTCAGCGTTTCAATTTTAGAT
2284            AGTTCTGCGAGGTTTGCTCTGTGATTATCAGTGAATGTATTCAGCGTTTCAATTTTAGAT
5814            AGTTCTGCGAGGTTTGCTCTGTGATTATCAGTGAATGTATTCAGCGTTTCAATTTTAGAT
HHV-6B.Z29      AGTTCTTCGAGGTTTGCTATGTGTTTATCAGTGAATGTATTTAGTATTTCAATTTTAGAT
HST             AGTTCTTCGAGGTTTGCTATGTGTTTATCAGTGAATGTATTTAGTATTTCAATTTTAGAT
                ****** *********** **** ************  *** **  **************
                                          U1102 indel-2    End ori-lyt(mori)68712
HHV-6A.U1102    TATGGTTCGGTAAAAATATAGGTCTTTGTAAAATCATTTGATTTAGCTTTTATATGTATT 68714
GS              TATGGTTCGGTAAAAATATAGGTCTTTG--------------------------------
AJ              TATGGTTCGGTAAAAATATAGGTCTTTG--------------------------------
CiHHV-6A.5055   TATGGTTCGGTAAAAATATAGGTCCTTG--------------------------------
2284            TATGGTTCGGTAAAAATATAGGTCTTTGTAAAATCATTTGATTTAGCTTTTATATGTATT
5814            TATGGTTCGGTAAAAATATAGGTCTTTGTAAAATCATTTGATTTAGCTTTTATATGTATT
HHV-6B.Z29      CATGGTTCGGTAAAGATATAAGTCCGTGTAAAATTTTTTGGTTTAGTTTTCATATCTACT
HST             CATGGTTCGGTAAAGATATAAGTCCGTGTAAAATTTTTTGGTTTAGTTTTCATATCTACT
                 ************* ***** ***  **
```

Figure S2. The origin of lytic replication, mori, of CiHHV-6A 5055/1623, 5814, and 2284/4305 compared to virus reference HHV-6A U1102, AJ, GS and HHV-6B Z29, HST.

References

1. Bell, A.J.; Gallagher, A.; Mottram, T.; Lake, A.; Kane, E.V.; Lightfoot, T.; Roman, E.; Jarrett, R.F. Germ-line transmitted, chromosomally integrated HHV-6 and classical Hodgkin lymphoma. *PLoS ONE* **2014**, *9*, e112642.
2. Faten, N.; Agnes, G.D.; Nadia, B.F.; Nabil, A.B.; Monia, Z.; Abderrahim, K.; Henri, A.; Salma, F.; Mahjoub, A. Quantitative analysis of human herpesvirus-6 genome in blood and bone marrow samples from Tunisian patients with acute leukemia: a follow-up study. *Infect. Agent Cancer* **2014**, *7*, 31, doi:10.1186/1750-9378-7-31.
3. Geraudie, B.; Charrier, M.; Bonnafous, P.; Heurte, D.; Desmonet, M.; Bartoletti, M.A.; Penasse, C.; Agut, H.; Gautheret-Dejean, A. Quantitation of human herpesvirus-6A, -6B and -7 DNAs in whole blood, mononuclear and polymorphonuclear cell fractions from healthy blood donors. *J. Clin. Virol.* **2012**, *53*, 151–155.
4. Gravel, A.; Sinnett, D.; Flamand, L. Frequency of chromosomally-integrated human herpesvirus 6 in children with acute lymphoblastic leukemia. *PLoS ONE* **2013**, *8*, e84322.
5. Hall, C.B.; Caserta, M.T.; Schnabel, K.; Shelley, L.M.; Marino, A.S.; Carnahan, J.A.; Yoo, C.; Lofthus, G.K.; McDermott, M.P. Chromosomal integration of human herpesvirus 6 is the major mode of congenital human herpesvirus 6 infection. *Pediatrics* **2008**, *122*, 513–520.
6. Hall, C.B.; Caserta, M.T.; Schnabel, K.C.; Boettrich, C.; McDermott, M.P.; Lofthus, G.K.; Carnahan, J.A.; Dewhurst, S. Congenital infections with human herpesvirus 6 (HHV6) and human herpesvirus 7 (HHV7). *J. Pediatr.* **2004**, *145*, 472–477.
7. Hubacek, P.; Hrdlickova, A.; Spacek, M.; Zajac, M.; Muzikova, K.; Sedlacek, P.; Cetkovsky, P. Prevalence of chromosomally integrated HHV-6 in patients with malignant disease and healthy donors in the Czech Republic. *Folia Microbiol.* **2013**, *58*, 87–90.
8. Hubacek, P.; Muzikova, K.; Hrdlickova, A.; Cinek, O.; Hyncicova, K.; Hrstkova, H.; Sedlacek, P.; Stary, J. Prevalence of HHV-6 integrated chromosomally among children treated for acute lymphoblastic or myeloid leukemia in the Czech Republic. *J. Med. Virol.* **2009**, *81*, 258–263.

9. Hudnall, S.D.; Chen, T.; Allison, P.; Tyring, S.K.; Heath, A. Herpesvirus prevalence and viral load in healthy blood donors by quantitative real-time polymerase chain reaction. *Transfusion* **2008**, *48*, 1180–1187.
10. Lee, S.O.; Brown, R.A.; Eid, A.J.; Razonable, R.R. Chromosomally integrated human herpesvirus-6 in kidney transplant recipients. *Nephrol. Dial. Transplant.* **2011**, *26*, 2391–3239.
11. Lee, S.O.; Brown, R.A.; Razonable, R.R. Chromosomally integrated human herpesvirus-6 in transplant recipients. *Transpl. Infect. Dis.* **2012**, *14*, 346–354.
12. Leong, H.N.; Tuke, P.W.; Tedder, R.S.; Khanom, A.B.; Eglin, R.P.; Atkinson, C.E.; Ward, K.N.; Griffiths, P.D.; Clark, D.A. The prevalence of chromosomally integrated human herpesvirus 6 genomes in the blood of UK blood donors. *J. Med. Virol.* **2007**, *79*, 45–51.
13. Potenza, L.; Barozzi, P.; Masetti, M.; Pecorari, M.; Bresciani, P.; Gautheret-Dejean, A.; Riva, G.; Vallerini, D.; Tagliazucchi, S.; Codeluppi, M.; *et al*. Prevalence of human herpesvirus-6 chromosomal integration (CIHHV-6) in Italian solid organ and allogeneic stem cell transplant patients. *Am. J. Transplant.* **2009**, *9*, 1690–1697.
14. Tanaka-Taya, K.; Sashihara, J.; Kurahashi, H.; Amo, K.; Miyagawa, H.; Kondo, K.; Okada, S.; Yamanishi, K. Human herpesvirus 6 (HHV-6) is transmitted from parent to child in an integrated form and characterization of cases with chromosomally integrated HHV-6 DNA. *J. Med. Virol.* **2004**, *73*, 465–473.
15. Tembo, J.; Kabwe, M.; Chilukutu, L.; Chilufya, M.; Mwaanza, N.; Chabala, C.; Zumla, A.; Bates, M. Prevalence and risk factors for betaherpesvirus DNAemia in infants aged between 3 weeks and 2 years of age, admitted to a large referral hospital in sub-Saharan Africa. *Clin Infect Dis.* **2014**, doi:10.1093/cid/ciu853.
16. Tweedy, J.; Spyrou, M.A.; Hubacek, P.; Kuhl, U.; Lassner, D.; Gompels, U.A. Analyses of germline, chromosomally integrated human herpesvirus 6A and B genomes indicate emergent infection and new inflammatory mediators. *J. Gen. Virol.* **2015**, *96*, 370–389.
17. Ward, K.N.; Andrews, N.J.; Verity, C.M.; Miller, E.; Ross, E.M. Human herpesviruses-6 and -7 each cause significant neurological morbidity in Britain and Ireland. *Arch. Dis. Child* **2005**, *90*, 619–623.
18. Ward, K.N.; Leong, H.N.; Thiruchelvam, A.D.; Atkinson, C.E.; Clark, D.A. Human herpesvirus 6 DNA levels in cerebrospinal fluid due to primary infection differ from those due to chromosomal viral integration and have implications for diagnosis of encephalitis. *J. Clin. Microbiol.* **2007**, *45*, 1298–1304.

© 2016 by the authors; licensee MDPI, Basel, Switzerland. This article is an open access article distributed under the terms and conditions of the Creative Commons by Attribution (CC-BY) license (http://creativecommons.org/licenses/by/4.0/).
